# Development of mechanical papillae of the tongue in the domestic goose (*Anser anser f. domestica*) during the embryonic period

**DOI:** 10.1007/s00709-015-0927-x

**Published:** 2015-12-21

**Authors:** Kinga Skieresz-Szewczyk, Hanna Jackowiak

**Affiliations:** Department of Histology and Embryology, Poznań University of Life Sciences, Wojska Polskiego 71 C, 60-625 Poznań, Poland

**Keywords:** Lingual papillae, Orthokeratinized epithelium, Embryonic development, Birds, Goose

## Abstract

Three types of mechanical papillae, i.e., conical, filiform, and hair-like papillae, are present on the tongue in the domestic goose. Within conical papillae, we distinguish three categories: large and small conical papillae on the body and conical papillae on the lingual prominence. The arrangement of mechanical papillae on the tongue in Anseriformes is connected functionally with different feeding mechanisms such as grazing and filter-feeding. The present work aims to determine whether morphology of three types of mechanical papillae in goose at the time of hatching is the same as in an adult bird and if the tongue is prepared to fulfill feeding function. Our results revealed that the primordia of the large conical papillae start to develop during the differentiation stage. The primordia of the small conical papillae and conical papillae of the lingual papillae start to develop during the growth stage. At the end of the growth stage, only large conical papillae, three pairs of small conical papillae, and conical papillae of the lingual prominence have similar arrangement as in an adult bird. The shape and arrangement of the remaining small conical papillae probably will be changed after hatching. During embryonic period, the filiform papillae and hair-like papillae are not formed. The embryonic epithelium that covered the mechanical papillae undergoes transformation leading to the formation of multilayered epithelium. During prehatching stage, epithelium becomes orthokeratinized epithelium. In conclusion, the tongue of the domestic goose after hatching is well prepared only for grazing. The filtration of food from water is limited due to the lack of filiform papillae.

## Introduction

The tongue in birds is characterized by significant variability between species in the range of size, shape, and type of lingual papillae, which are largely related to the type of food and the mechanism of food intake (Von Preuss et al. 1969; McLelland 1979, 1990; Iwasaki and Kobayashi 1986; Kooloos 1986; Homberger and Meyers 1989; Vollmerhaus and Sinowatz 1992; Iwasaki et al. 1997a, b; Zweers et al. 1997; Van Der Leeuw et al. 2003; Rossi et al. 2005; Jackowiak and Godynicki 2005; Glatz et al. 2006; Jackowiak et al. 2010, 2011).

The lingual papillae fulfill diverse functions during food intake, transport, and swallowing. In most avian species, only one type of mechanical papillae has been found, such as conical papillae located on the border between the lingual body and root of the tongue (Iwasaki and Kobayashi 1986; Homberger and Meyers 1989; Jackowiak and Godynicki 2005; Rossi et al. 2005; Emura and Chen 2008; Emura et al. 2008, 2009, 2010; Igwebuike and Ukamaka, 2010; Parchami and Dehkordi 2011; Igwebuike and Anagor 2013). Those papillae have the shape of cones arranged individually in one or two rows or grow from common crest (Iwasaki and Kobayashi 1986; Jackowiak and Godynicki 2005; Rossi et al. 2005; Jackowiak et al. 2010; Kadhim et al. 2011; Parchami and Dehkordi 2011). The tips of the conical papillae are directed towards the pharynx. The conical papillae perform functions connected with retaining the food in the oral cavity and directing food into the esophagus. A unique arrangement of conical papillae is observed in penguins. Conical papillae in the form of spikes are present along the entire dorsal surface of the tongue, and their function is to hold the fish and transport them into the esophagus (Kobayashi et al. 1998).

In Anseriformes, conical papillae are located not only on the border of lingual prominence and root of the tongue but also are present on the edges of the lingual body (McLelland 1979, 1990; Nickel et al. 1992; Iwasaki et al. 1997a, b; Hassan et al. 2010; Jackowiak et al. 2011; Igwebuike and Anagor 2013). The function of the conical papillae on the body is grazing blades of grass in cooperation with the lamellae in the beak (Jackowiak et al. 2011). In Anseriformes, there are two additional types of mechanical papillae, namely filiform papillae present in the form of long appendages between conical papillae on the lingual body and hair-like papillae present on the dorsolateral surface of the caudal part of the body of the tongue (Iwasaki et al. 1997a, b; Jackowiak et al. 2011; Igwebuike and Anagor, 2013). The conical papillae of the body of the tongue and filiform papillae together form the so-called filtration apparatus, which functions to filter food particles from water (Jackowiak et al. 2011; Skieresz-Szewczyk and Jackowiak 2014).

The microscopic studies in birds indicate that the lingual mucous is covered with the para- and orthokeratinized epithelium (Iwasaki et al. 1997a, b; Skieresz-Szewczyk et al. 2014a, b). Because of their resistance functions, conical papillae are covered with orthokeratinized epithelium which in birds is composed of the basal layer, an intermediate layer divided into lower and upper parts and the keratinized layer (Iwasaki et al. 1997a, b; Jackowiak et al. 2011; Skieresz-Szewczyk et al. 2014a, b). Cells in the intermediate and keratinized layers exhibit characteristics of β-keratinization without producing keratohyalin granules (Alibardi 2004). In turn, filiform papillae and hair-like papillae are the only keratinized processes of the epithelium (Jackowiak et al. 2011; Skieresz-Szewczyk and Jackowiak 2014).

The present literature on the embryogenesis of the tongue in birds and development of lingual papillae only provides information concerning the early development of the tongue in the chicken and further differentiation of some morphological structures of the tongue in the chicken, goose, and duck (Lillie 1908; Bryk et al. 1992; Skieresz-Szewczyk et al. 2012, 2014a, b).

Distribution of the three types of mechanical papillae in the goose is connected with food intake by grazing and filter-feeding and also with food transport (Van Der Leeuw et al. 2003; Zweers et al. 1997). In the present work, it was decided to verify the following research hypothesis: the morphology of the lingual papillae and their arrangement on the tongue in the domestic goose at the time of hatching is similar to that in the adult bird. Thus, the tongue is ready to perform the abovementioned food activities.

In order to verify the hypothesis, the formation of the lingual papillae primordia, their differentiation, the rate of their development and changes in the shape and arrangement were analyzed using a scanning electron microscope. The purpose of light microscope examination is to determine changes in the histological structure of the epithelium covering the lingual papillae.

## Material and methods

A total of 68 fertilized eggs of the domestic goose were incubated in a breeding chamber at a temperature of 37.8 °C, relative humidity 50–55 %, and the composition of air 2 % O_2_ and 0.4 % CO_2_ at the Poultry Hatching Facility in Gniezno, Poland. For the purpose of the study, four eggs of the domestic goose were collected at 24-h intervals between the 9th and 25th day of incubation. After being removed from the breeding chamber, eggs were placed at a temperature of 4 °C for 4 h.

Embryos were removed from eggs, decapitated, rinsed in saline and subsequently fixed in 4 % buffered formalin for 48 h. Two entire tongues were dissected and prepared for LM by dehydration in an ascending series of ethanol (70–96 %) and routinely embedded in paraplast. Histological slides of approximately 4.5 μm in thickness were stained according to the Masson-Goldner trichrome method. The results were documented using an Axioscope 2 plus light microscope (Zeiss, Germany). Two other entire tongues were dissected and prepared for SEM observations by dehydration in a series of ethanol (70–99.8 %) for 15 min, then in a mixture of 96 % ethanol and 100 % acetone for 10 min, and in 100 % acetone for 5 min. The tongues were then dried at critical point using CO_2_ (Critical Point Dryer K850, EMITECH). All samples were mounted on aluminum stubs covered with carbon tabs, sputtered with gold (Sputter Coater S 150B, EDWARDS), and observed under the SEM LEO 435 VP microscope (ZEISS) at an accelerating voltage of 10–15 kV. The length and width of the mechanical papillae and angles of inclination of the conical papillae of the body to the long axis of the tongue were measured by software supporting the SEM LEO 435 VP (ZEISS) microscope. The recorded measurements of the height and width of mechanical papillae were statistically tested using the Statistica 10.0 software. Mean values (*X*) with standard deviation (SD) were calculated for each morphological feature.

The study was conducted in accordance with the approval and guidelines set out by the Ethics Commission at the Poznan University of Life Sciences in Poland.

Based on our observations of domestic goose embryos and on the studies of Hamburger and Hamilton (1951) and Koecke (1958), Hamburger-Hamilton (HH) developmental stages are assigned to the particular days of incubation.

## Results

The tongue as an elongated organ with flat mucosa is observed until the tenth day of incubation (33/35 HH stage).

The morphometric analysis of the lingual papillae during the embryonic period is summarized in Table 1.

**Table 1:** Measurements of the height and diameter of the conical papillae during embryonic development in the domestic goose

Day of incubation	Large conical papillae		Small conical papillae		Conical papillae of the lingual prominence	
	Height (μm) *X* ± SD	Diameter (μm) *X* ± SD	Height (μm) *X* ± SD	Diameter (μm) *X* ± SD	Height (μm) *X* ± SD	Diameter (μm) *X* ± SD
9	–	–	–	–	–	–
10	–	–	–	–	–	–
11	79.8 ± 7.6	94.4 ± 14.3	–	–	–	–
12	83.2 ± 8.3	97.3 ± 14.7	–	–	–	–
13	89.5 ± 51.7	105.2 ± 26.2	–	–	–	–
14	151.8 ± 60.0	102.5 ± 42.9	–	–	–	–
15	178.3 ± 112.1	169.0 ± 14.6	–	–	–	–
16	297.7 ± 76.1	163.1 ± 8.1	224.2 ± 29.9	160.0 ± 12.2	102.0 ± 25.1	57.3 ± 1.5
17	349.8 ± 62.0	252.9 ± 16.1	116.2 ± 38.0	130.9 ± 18.5	123.2 ± 8.8	65.4 ± 13.9
18	684.1 ± 58.8	272.8 ± 23.0	129.5 ± 39.3	200.2 ± 17.8	221.2 ± 24.8	135.0 ± 44.2
19	619.4 ± 39.7	225.4 ± 23.1	141.8 ± 27.0	175.4 ± 20.7	271.6 ± 20.2	170.3 ± 79.1
20	748.1 ± 160.5	269.1 ± 24.0	142.0 ± 25.9	172.6 ± 22.0	279.8 ± 40.8	213.9 ± 79.1
21	858.8 ± 162.4	301.5 ± 37.2	255.1 ± 25.9	211.5 ± 27.6	401.4 ± 87.0	194.6 ± 51.0
22	841.0 ± 109.5	324.7 ± 27.7	255.8 ± 17.3	200.0 ± 35.2	398.6 ± 49.1	212.5 ± 35.9
23	848.7 ± 107.6	259.3 ± 18.8	237.4 ± 22.4	180.1 ± 13.4	319.6 ± 51.4	243.2 ± 58.1
24	903.8 ± 241.0	283.1 ± 61.6	203.1 ± 14.5	204.3 ± 22.8	294.9 ± 50.6	356.9 ± 43.0
25	1207.2 ± 170.0	385.1 ± 30.8	282.5 ± 12.4	222.7 ± 16.7	423.1 ± 41.7	308.3 ± 59.6

### Conical papillae of the lingual body

#### Between the 11th and 13th day of incubation (36–38 HH stage)

**Fig. 1:**
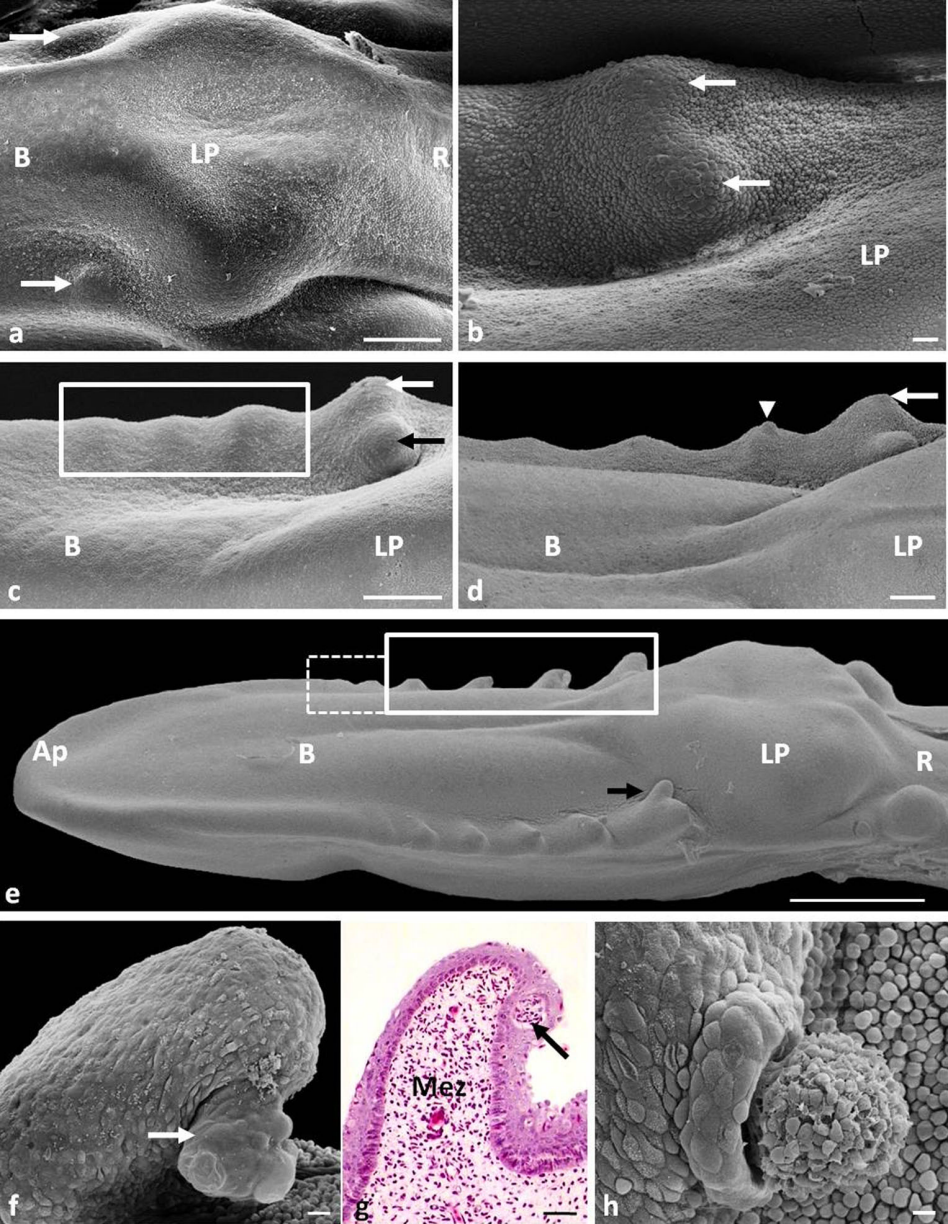
**a** Eleventh day of incubation. Dorsal view on the posterior part of the lingual body and lingual prominence. *White arrows* point to the primordia of the first pair of large conical papillae. *B* body of the tongue, *LP* lingual prominence, *R* root of the tongue. SEM. Scale bar 300 μm. **b** Twelfth day of incubation. Dorsal view on the lateral part of the lingual body. *White arrows* show two parts of the first large conical papillae. *LP* lingual prominence. SEM. Scale bar 20 μm. **c** Thirteenth day of incubation. Dorsal view on the lateral part of the lingual prominence. *Rectangle as continuous line* points to three new primordia of large conical papillae. *White arrow* shows the lateral part of the first large conical papillae. *Black arrow* points to the medial part of the first large conical papillae. *B* body of the tongue, *LP* lingual prominence. SEM. Scale bar 300 μm. **d** Fifteenth day of incubation. Lateral view on the four primordia of triangular-shaped large conical papillae. *White arrows* points to larger lateral parts of the first large conical papillae. *Arrow head* shows the spherical structure on the tip of papillae. *B* body of the tongue, *LP* lingual prominence. SEM. Scale bar 100 μm. **e** Sixteenth day of incubation. Lateral view on the tongue. *Rectangle as continuous line* points to large conical papillae. *Rectangle as dashed line* shows the primordia of small conical papillae. The *black arrow* points to rounded medial parts of the first large conical papillae. *Ap* apex of the tongue, *B* body of the tongue, *LP* lingual prominence, *R* root of the tongue. SEM. Scale bar 1 mm. **f** Eighteenth day of incubation. Magnification of large conical papillae with spherical structure (*white arrow*). SEM. Scale bar 20 μm. **g** Eighteenth day of incubation. Cross-section through large conical papillae. *White arrow* shows spherical structure with its own mezenchymal core. LM. Scale bar 20 μm. **h** Eighteenth day of incubation. Magnification of the degenerate spherical structure. SEM. Scale bar 10 μm

On the 11th day, round primordia of the first pair of large conical papillae, it is two primordia, one primordium on the right side and one primordium on the left side of the lingual body, are formed in front of the lingual prominence, in recesses of the caudolateral surface (Fig. 1a). The primordia are elevations of the mesenchymal tissue (Fig. 3a).

**Fig. 2:**
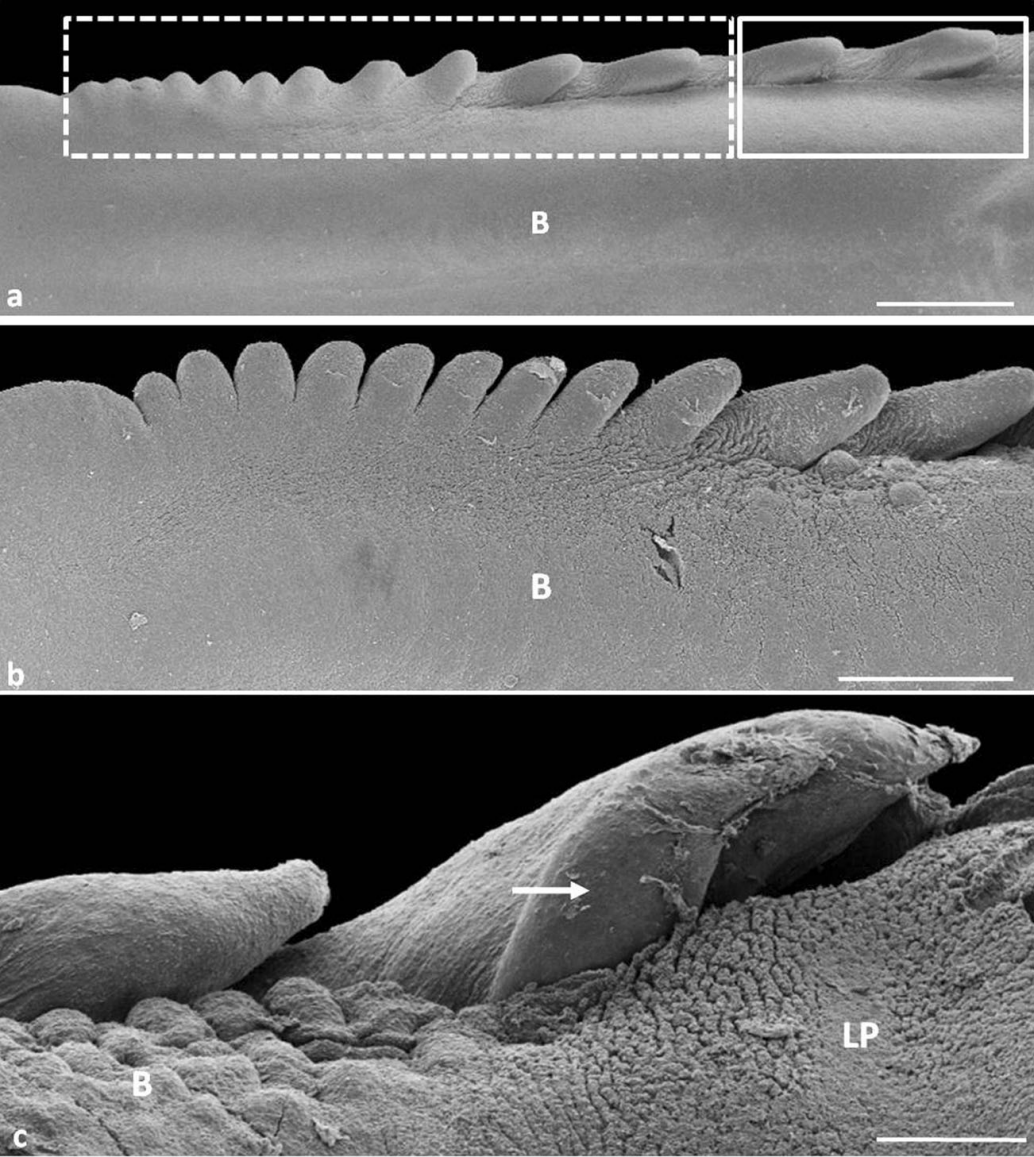
**a** Eighteenth day of incubation. Dorsal view on the lateral part of the lingual body. *Rectangle as continuous line* points to large conical papillae. *Dashed line* shows small conical papillae. *B* body of the tongue. SEM. Scale bar 1 mm. **b** Twenty-first day of incubation. Dorsal view on the lateral part of the lingual body with small conical papillae. *B* body of the tongue. SEM. Scale bar 1 mm. **c** Twenty-third day of incubation. Magnification of the first and second large conical papillae. *White arrows* points to smaller, sharpened medial parts of the first large conical papillae. *B* body of the tongue, *LP* lingual prominence. SEM. Scale bar 300 μm

**Fig. 3:**
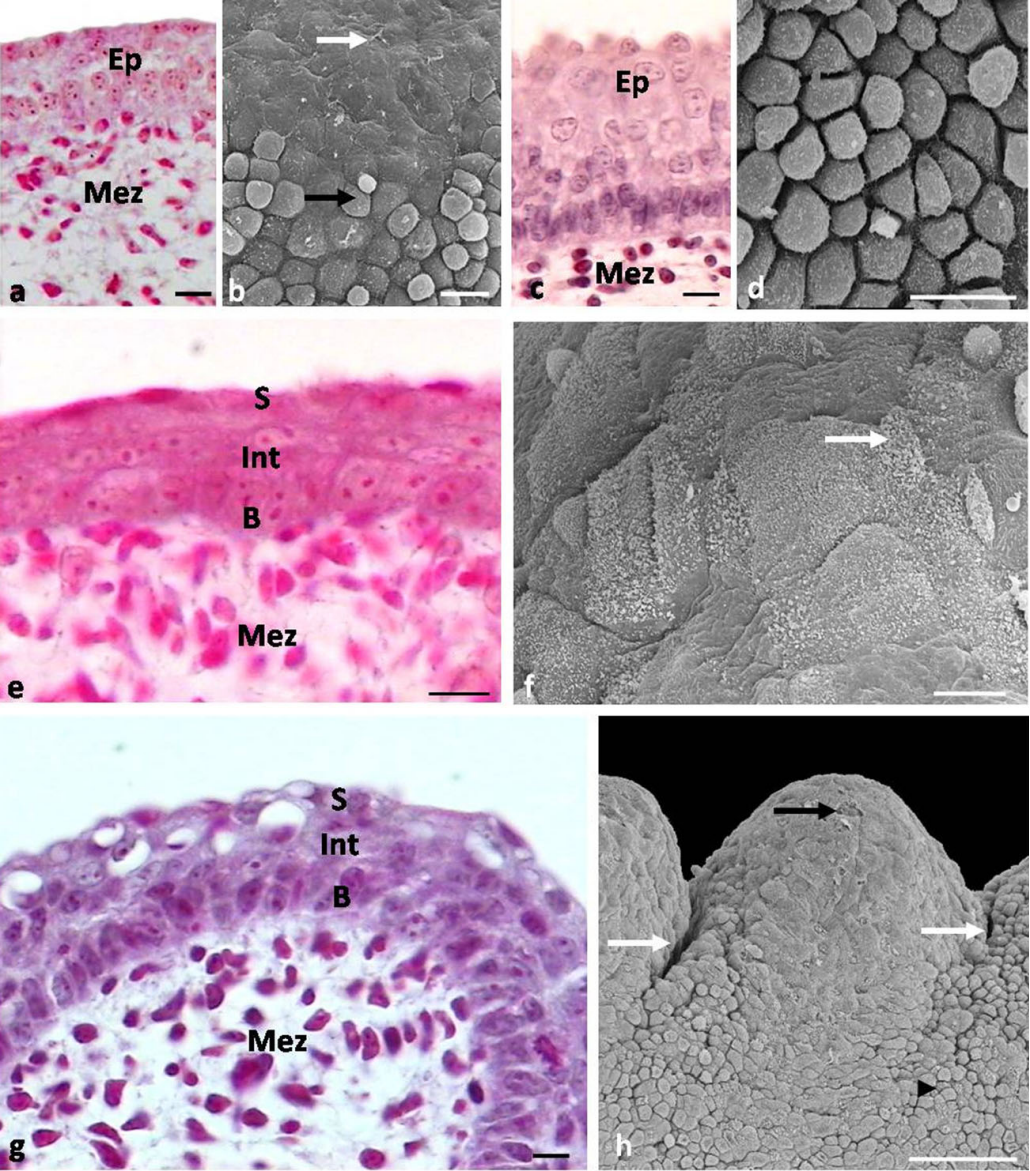
**a** Eleventh day of incubation. Magnification of embryonic epithelium of large conical papillae. *Ep* embryonic epithelium, *Mez* mesenchyme. LM. Scale bar 10 μm. **b** Eleventh day of incubation. Magnification of the tip of large conical papillae. *White arrow* shows a flat superficial cell. *Black arrow* points to a rounded and convex superficial cell. SEM. **c** Sixteenth day of incubation. Cross-section through the epithelium of the small conical papillae. *Ep* embryonic epithelium, *Mez* mesenchyme. LM. Scale bar 10 μm. **d** Sixteenth day of incubation. Magnification of the epithelium on the tip of the small conical papillae with rounded superficial cells. SEM. Scale bar 10 μm. **e** Sixteenth day of incubation. Cross-section through the epithelium of large conical papillae. *B* basal layer, *Int* intermediate layer, *Mez* mesenchyme, *S* superficial layer. LM. Scale bar 10 μm. **f** Sixteenth day of incubation. Magnification of superficial cells of epithelium on the tip of large conical papillae. *White arrow* points to microvilli. SEM. Scale bar 10 μm. **g** Eighteenth day of incubation. Cross-section through the epithelium of small conical papillae. *B* basal layer, *Int* intermediate layer, *Mez* mesenchyme, *S* superficial layer. LM. Scale bar 10 μm. **h** Eighteenth day of incubation. Magnification of small conical papillae. *White arrows* point to grooves separating papillae. *Black arrow* shows exfoliated superficial cell. *Arrow head* points to embryonic epithelium at the basis of papillae. SEM. Scale bar 100 μm

On the 12th day of incubation, a small groove is found in the central part of the first pair of papillae primordia, and the primordia are divided into two parts (Fig. 1b).

One day later, there are three pairs of new primordia of large conical papillae in the form of slightly elongated or rounded elevations of the mesenchymal tissue and therefore on the lingual body all four pairs of large conical papillae are visible (Fig. 1c). The primordia of the first pair of large conical papillae are clearly divided into two rounded and equally sized parts: the medial and lateral (Fig. 1c). The medial part of the primordia is located lower than the lateral part. All primordia of large conical papillae are arranged at an angle of 80°–90° in relation to the long axis of the tongue.

Between the 11th and 13th day, the tips of large conical papillae are covered with embryonic epithelium built of 3–4 layers of cells (Fig. 3a). Superficial cells on the tips of primordia are round in outline and slightly flattened (Fig. 3b). The embryonic epithelium on the sides of the papillae core is composed of many layers of cells, which number increases towards the base of the papillae (Fig. 3a). Superficial cells on sides of primordia are round and convex (Fig. 3b). A number of cell microvilli are observed on the cell surface.

#### 14th and 15th days of incubation (39/40 HH stage)

No new primordia of large conical papillae are formed. The primordia of large conical papillae on the 15th day change their shape from rounded to triangular (Fig. 1d). The lateral part of the first pair of primordia of large conical papillae is larger than the medial part (Fig. 1d). The spherical structures are present at the tip of the first and second pairs of large conical papillae (Fig. 1d).

#### 16th day of incubation (40 HH stage)

The primordia of the four pairs of large conical papillae are conical in shape with rounded tips (Fig. 1e). The exception is the medial part of the primordium of the first pair of conical papillae, which is still rounded (Fig. 1e). Additionally, the papillae change their position and now are directed slightly caudally at an angle of 50°–60° to the long axis of the tongue. Spherical structures are observed on the caudal surface of the primordium of the first and second pairs of large papillae (Fig. 1f). Their diameter is about 63 μm.

The epithelium at the tip of large conical papillae at the 16th day of incubation is built of 4–5 layers of cells (Fig. 3e). The basal, intermediate, and superficial layers are distinguished in the epithelium (Fig. 3e).

The cells in the basal layer, similarly as their nuclei, are oval (Fig. 3e). The intermediate layer is formed by polygonal cells with oval nuclei arranged horizontally (Fig. 3e). The cells in the superficial layer are flat, and their nuclei contain condensed chromatin (Fig. 3e). Observations of the epithelium surface show that the cells are polygonal, while the nuclei of some cells are convex (Fig. 3f). The cell surface reveals the presence of microvilli (Fig. 3f).

At the 16th day, the primordia for the three pairs of small conical papillae are also formed (Fig. 1e). The primordia of the first and second pairs of small conical papillae are slightly elongated and arranged at an angle of 65°–70° to the long axis of the tongue. The primordia of the third pair of small conical papillae form a rounded elevation of the mesenchymal tissue located to the sides of the lingual body at an angle of 90°.

The small conical papillae are covered with a multilayer embryonic epithelium, which consists of 7–8 layers of cells (Fig. 3c). Superficial cells are round and convex (Fig. 3d).

#### 17th day of incubation (41 HH stage)

There are no signs of new primordia of small conical papillae. The primordia of the first pair of small conical papillae take the shape of cones with rounded tops, arranged at an angle of 50° to the long axis of the tongue. Two further pairs of primordia of small conical papillae are triangular and arranged at an angle of 60°.

#### 18th day of incubation (41/42 HH stage)

On the edges of the lingual body, four pairs of large conical papillae and nine pairs of small conical papillae are visible. At that day, the large conical papillae are arranged at an angle of 20°–25° to the long axis of the tongue. Figure 1g clearly shows that the spherical structure has its own core consisting of the mesenchymal tissue, covered with epithelium. The spherical structure starts to degenerate and break away from the papillae (Fig. 1h).

At the 18th day, cores of the first two pairs of small conical papillae are clearly separated from each other and arranged at an angle of 35°–40° to the long axis of the tongue (Fig. 3h). The primordia of the third, fourth, fifth, and sixth pairs of small conical papillae are located at an angle of 55°, and the other three, rounded pairs of primordia of small conical papillae are arranged at an angle of 80°–90°.

The structure of epithelium covering the small conical papilla changes at the 18th day of incubation. The epithelium is built of 4–5 layers of cells, and the basal, intermediate, and superficial layers are distinguished (Fig. 3g). The layers are structurally similar to those in the epithelium of the large conical papillae. Single superficial cells exfoliate (Fig. 3h).

#### 19th day of incubation (41/42 HH stage)

There are new primordia of two pairs of small conical papillae, and thus on the lingual body, there are four pairs of large conical papillae and eleven pairs of small conical papillae. At the 19th day of incubation, a third pair of small conical papillae is conical in shape with a rounded tip, and together with the first and second pairs of small conical papilla, they are arranged at an angle of 30° to the long axis of the tongue (Fig. 2a). The small conical papillae, from four to eight pairs, are triangular and arranged at an angle of 50° (Fig. 2a). The other small conical papillae are rounded and arranged at an angle of 80°–90° (Fig. 2a). Spherical structures on the caudal part of large and small conical papillae are no longer present.

### Between 20th and 25th day of incubation (43–45 HH stage)

Around the 20th/21st day, the small conical papillae from the 4th to 11th pairs take a finger-like shape and their cores are separated (Fig. 2b). The papillae from the 4th to the 8th pairs are arranged at an angle of 40°–45°, and from 9th to 11th pairs at an angle of 80°–90° (Fig. 2b).

**Fig. 4:**
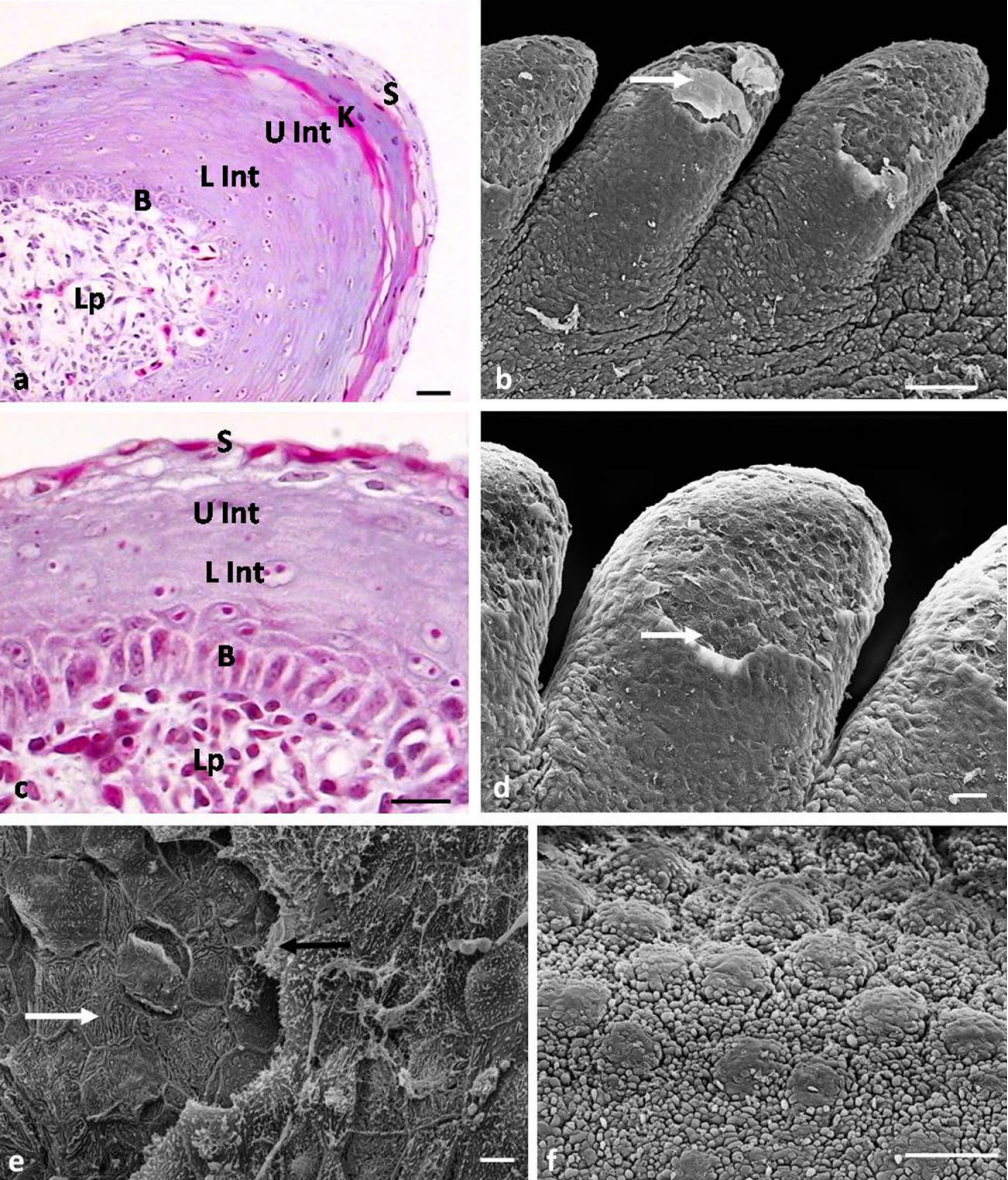
**a** Twenty-first day of incubation. Cross-section through the epithelium of large conical papillae. *B* basal layer, *L Int* lower part of the intermediate layer, *Lp* lamina propria, *K* keratinized layer, *U Int* upper part of the intermediate layer, *S* superficial layer. LM. Scale bar 10 μm. **b** Twenty-first day of incubation. Magnification of large conical papillae. *White arrow* shows exfoliated superficial cell. SEM. Scale bar 100 μm. **c** Twenty-first day of incubation. Cross-section through the epithelium of small conical papillae. *B* basal layer, *L Int* lower part of the intermediate layer, *Lp* lamina propria, *U Int* upper part of the intermediate layer, *S* superficial layer. LM. Scale bar 10 μm. **d** Twenty-first day of incubation. Magnification of small conical papillae. *White arrow* shows exfoliated superficial cell. SEM. Scale bar 30 μm. **e** Twenty-third day of incubation. Magnification of the epithelium of large conical papillae. *White arrow* points to microridges. *Black arrow* shows exfoliated superficial cell. SEM. Scale bar 10 μm. **f** Twenty-first day of incubation. Magnification of bulges on the dorsolateral surface of the caudal part of the lingual body. SEM. Scale bar 100 μm

At the 21st day of incubation, the structure of the epithelium at the first pair of large conical papillae is changed (Fig. 4a). The epithelium is built up of 19–20 layers of cells.

The structure of the basal layer is similar to the previous day of development. In the intermediate layer, two parts are distinguished. The lower part of the intermediate layer is composed of polygonal cells with oval nuclei arranged horizontally (Fig. 4a). The cytoplasm of these cells, as shown in the Masson-Goldner staining, is dyed bright pink. Cells in the upper part of the intermediate layer are polygonal, but their nuclei are flat, and the cytoplasm is stained dark pink (Fig. 4a). The keratinized layer of the large conical papillae is composed of two cell layers, which are highly flattened cells without cell nuclei (Fig. 4a). The cytoplasm of cells of the keratinized layer, after Masson-Goldner staining, is heavily stained red. Over the keratinized layer, we observed a surface layer consisting of 5–6 layers of polygonal cells, which, next to the keratinized layer, have oval nuclei with condensed chromatin (Fig. 4a). Figure 4b is made using SEM, and this indicates that the cells on the surface of the large conical papillae undergo massive exfoliation.

At the 21st day, the number of cell layers building the epithelium of small conical papillae increases up to 12–13 layers (Fig. 4c). The structure of the intermediate layer is also changed, and similarly, as in the epithelium of large conical papillae, it is divided into two parts, the lower and upper (Fig. 4c). The cells in the superficial layer are flat, and their nuclei have condensed chromatin (Fig. 4c). The cells on the surface of the epithelium have an intensely stained cell cytoplasm. Observations of the epithelium surface show that the cells are strongly flattened, polygonal, and exfoliated (Fig. 4d).

Between the 22nd and 25th day of incubation, the number, shape, and arrangement of the large and small conical papillae to the long axis of the tongue do not change. Figure 2c reveals that the medial part of the first pair of large conical papillae is still smaller than the lateral part. Both parts have sharpened tips.

At the 22nd day, the keratinized layer is formed on the epithelium of the second pair of large conical papillae. A day later, the 23rd day, a keratinized layer is formed on the third pair, and at the 24th day, it is observed on all large conical papillae. In the studied embryonic period, the keratinized layer is covered with layers of superficial cells. Observations of the epithelium surface of the large conical papillae in SEM at the 23rd day of incubation show that the epithelium at the tip of papillae peels off and the cells are flat and polygonal (Fig. 4e). Numerous microridges are observed on the surface of the keratinized cells (Fig. 4e).

The epithelium structure of the small conical papillae between the 22nd and 25th day does not change when compared to the previous days.

### Conical papillae of the lingual prominence

#### 16th day of incubation (40 HH stage)

**Fig. 5:**
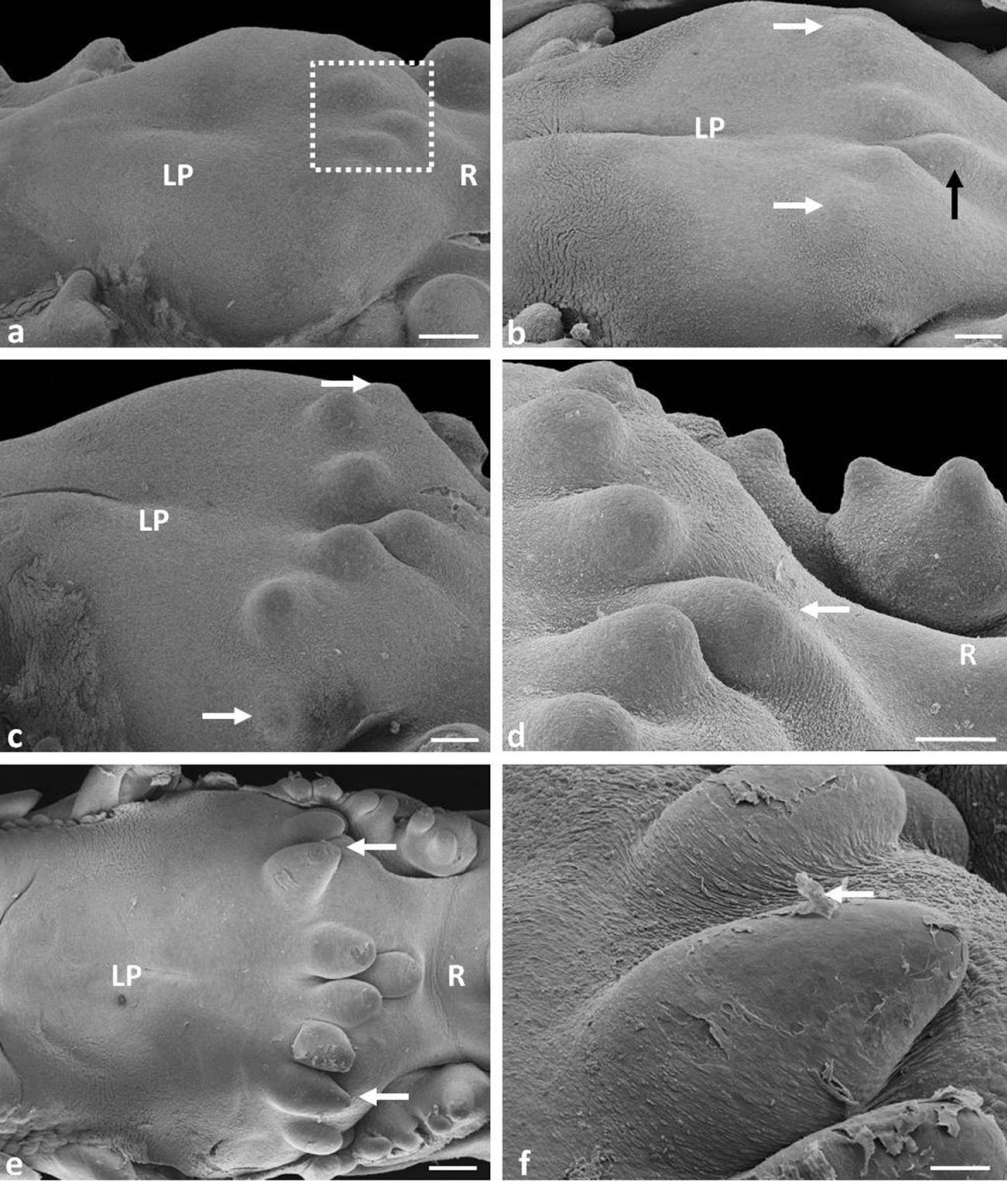
**a** Sixteenth day of incubation. Lateral view on lingual prominence. *Square as dotted line* points to the primordia of the three conical papillae of lingual prominence. *LP* lingual prominence, *R* root of the tongue. SEM. Scale bar 300 μm. **b** Seventh day of incubation. Lateral view on lingual prominence. *White arrows point* to two new primordia of conical papillae of lingual prominence. *Black arrow* shows median conical papillae shifted to the root of the tongue. *LP* lingual prominence. SEM. Scale bar 100 μm. **c** Eighteenth day of incubation. Lateral view on lingual prominence. *White arrows* point to two new primordia of conical papillae of lingual prominence. *LP* lingual prominence. SEM. Scale bar 300 μm. **d** Nineteenth day of incubation. Lateral view on lingual prominence. *White arrow* points to median conical papillae located below other conical papillae. *R* root of the tongue. SEM. Scale bar 300 μm. **e** Twenty-third day of incubation. Dorsal view on lingual prominence. *White arrow* points to conical papillae with tapered tips. *LP* lingual prominence, *R* root of the tongue. SEM. Scale bar 300 μm. **f** Twenty-third day of incubation. Magnification of conical papillae of lingual prominence. *White arrow* points to exfoliated superficial cells. SEM. Scale bar 100 μm

**Fig. 6:**
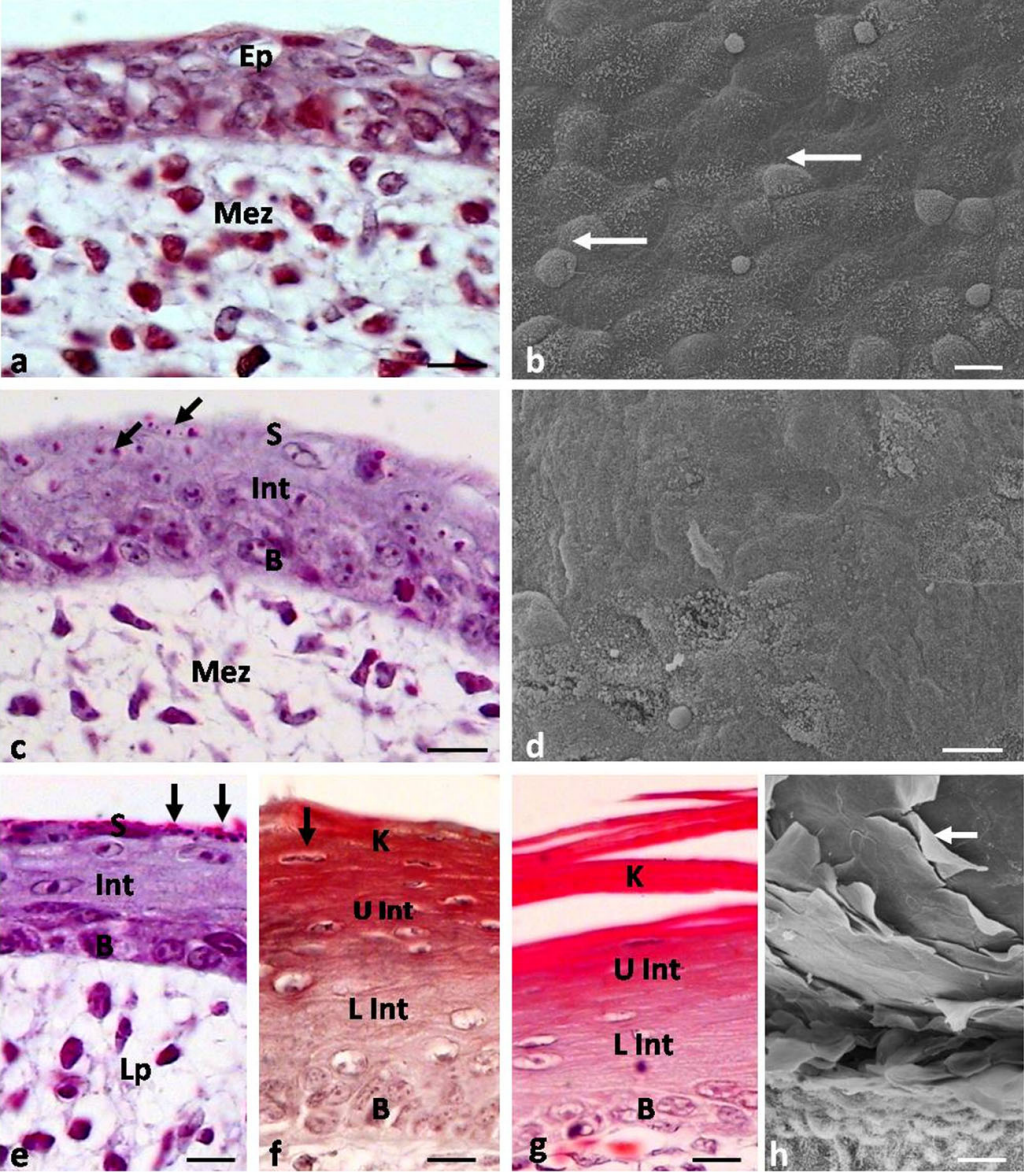
**a** Sixteenth day of incubation. Cross-section through the embryonic epithelium of conical papillae of lingual prominence. *Ep* epithelium, *Mez* mezenchyme. LM. Scale bar 10 μm. **b** Sixteenth day of incubation. Magnification of the epithelium on tips of conical papillae of lingual prominence. *White arrows* point to rounded, convex superficial cells. SEM. Scale bar 10 μm. **c** Eighteenth day of incubation. Cross-section through the epithelium of conical papillae of the lingual prominence. *Black arrows* point to red granules in the superficial layer. *B* basal layer, *Int* intermediate layer, *Mez* mezenchyme, *S* superficial layer. LM. Scale bar 10 μm. **d** Eighteenth day of incubation. Magnification of the epithelium on tips of conical papillae of the lingual prominence with polygonal superficial cells. SEM. Scale bar 10 μm. **e** Nineteenth day of incubation. Cross-section through the epithelium of conical papillae of the lingual prominence. *Black arrows* point to red granules in the superficial layer. *B* basal layer, *Int* intermediate layer, *Lp* lamina propria, *S* superficial layer. LM. Scale bar 10 μm. **f** Twenty-third day of incubation. Cross-section through the epithelium of conical papillae of the lingual prominence. *Black arrow* points to condensed cell nuclei. *B* basal layer, *L Int* lower part of the intermediate layer, *K* keratinized layer, *U Int* upper part of the intermediate layer. LM. Scale bar 10 μm. **g** Twenty-fifth day of incubation. Cross-section through the epithelium of conical papillae of the lingual prominence. *B* basal layer, *L Int* lower part of the intermediate layer, *K* keratinized layer, *U Int* upper part of the intermediate layer. LM. Scale bar 10 μm. **h** Twenty-fifth day of incubation. Magnification of the epithelium of conical papillae of the lingual prominence. *White arrow* points to exfoliated superficial cell. SEM. Scale bar 10 μm

On the postero-median part of the lingual prominence, three rounds of primordia of the conical papillae of the lingual prominence are formed as an elevation of mesenchymal tissues (Figs. 5a and 6a). One primordium, arranged in the median part of the lingual prominence, is shifted slightly caudally, and two remaining primordia are arranged on the sides in a straight line across the lingual prominence (Fig. 5a).

The embryonic epithelium covering the conical papillae of the lingual prominence is built of 3–4 cell layers (Fig. 6a). SEM observations of the epithelial surface show that the cells are round in shape and in some cells convex nuclei are visible (Fig. 6b). On the cell surface, a number of microvilli are seen.

#### 17th day of incubation (41 HH stage)

Two new round primordia of the conical papillae in the form of small mesenchymal elevation are formed. The primordia are arranged on the sides of the existing papillae, and in this way, five primordia of conical papillae are visible on the lingual prominence (Fig. 5b). The first primordium of the median conical papillae is clearly shifted to the root of the tongue and is separated from the two adjacent papillae by longitudinal grooves on both sides of the primordium (Fig. 5b).

#### 18th day of incubation (41/42 HH stage)

Two new primordia in the form of round mesenchymal elevations are observed, and on the lingual prominence, seven conical papillae are present (Fig. 5c). Cores of the previously formed primordia of the conical papillae are separated. The tips of papillae are rounded.

The microscopic observations of the epithelium made at days 17 and 18 showed that the epithelium is composed of 4–5 layers of cells (Fig. 6c). The basal, intermediate, and superficial layers are distinguished. The cells in the basal layer are oval with oval nuclei. The cells in the intermediate layer are polygonal and have oval, horizontally arranged nuclei. SEM images reveal that superficial cells are polygonal in shape and flattened, while some of them are wrinkled (Fig. 6d). Microvilli are still visible on the cell surface. The histological section through the conical papillae of the lingual prominence shows that the cytoplasm of the superficial cells does not have cell nuclei (Fig. 6c). The cytoplasm of cells after Masson-Goldner trichrome staining is pink in color and single red granules are visible (Fig. 6c).

#### 19th day of incubation (41/42 HH stage)

Nine primordia of conical papillae are present on the lingual prominence. The two newest primordia of conical papillae are round in shape. The other primordia are slightly extended and their tips are rounded (Fig. 5d). The first primordium of the median conical papillae of the lingual prominence is located below other conical papillae (Fig. 5d).

At that day, the epithelium of the conical papillae of the lingual prominence is built of 6–7 layers of cells (Fig. 6e). The structure of the basal and intermediate layer is the same as in the previous days. In the cytoplasm of the surface layer, numerous red granules are observed (Fig. 6e).

### Between 20th and 22nd day of incubation (43/44 HH stage)

At those days, no new primordial of conical papillae are formed. The shape of the already existing papillae does not change.

### 23rd day of incubation (44/45 HH stage)

The last primordium of the conical papillae of the lingual prominence is formed. An important finding of this day is the changing shape of some primordia of the conical papillae, which tips are narrow and are slightly tapered (Fig. 5e).

The epithelium of the conical papillae of the lingual prominence at day 23 is flat, multilayered with the keratinized layer, and is formed of 15–16 layers of cells (Fig. 6f). The cells in the basal layer are cylindrical with elongated or rounded cell nuclei (Fig. 6f). The intermediate layer is composed of two parts. The lower part is formed by polygonal cells with oval nuclei, arranged horizontally (Fig. 6f). Most cells in the upper part are devoid of cell nuclei and the cytoplasm, after Masson-Goldner staining, is dark pink (Fig. 6f). The keratinized layer is built of flat cells, which nuclei have highly condensed chromatin (Fig. 6f). The cytoplasm of these cells has an intense red color. The superficial cells exfoliate (Fig. 5f).

### Between 24th and 25th day of incubation (45 HH stage)

All primordia of the conical papillae of the lingual prominence are narrow, and the tips take the shape of slightly elongated, sharpened cones.

At the 25th day of incubation, cell nuclei in the keratinized layer are absent, and the cytoplasm, after Masson-Goldner staining, has an intense red color (Fig. 6g). The superficial cells massively peel off (Fig. 6h).

### Hair-like papillae and filiform papillae

#### Between 21st and 25th day of incubation (43–45 HH stage)

At the 21st day, on the dorsolateral surface of the caudal part of the lingual body, immediately before the lingual prominence, where hair-like papillae are present in adults, three rows of round bulges of the epithelium are formed (Fig. 4f). In the next days, the shape and the number of epithelium bulges do not change.

## Discussion

Deeming and Ferguson (1991) distinguish two stages of development in the embryonic development of birds, the differentiation stage from 1 to 38 HH and the growth stage from 39 HH to hatching. In the studied domestic goose, the differentiation stage occurs between the 1st and 13th day of incubation and the growth stage between the 14th and 25th day of incubation.

In the differentiation stage, the primordium of the tongue is formed. The rostral two-thirds of the tongue in birds is formed by merging the tuberculum impar and the right and left lateral lingual prominences, and the caudal one-third of the tongue is formed from copula (Lillie 1908). This study revealed that during the differentiation stage in the goose until the tenth day of incubation (33/35 HH stage), the dorsal surface of the tongue is smooth. Studies on the morphogenesis of the avian tongue have shown that in chickens until the seventh day (31 HH stage) and in the duck until the ninth day of incubation (32/33 HH stage), the mucous membrane of the tongue is also smooth (Bryk et al. 1992; Skieresz-Szewczyk et al. 2014a, b). In the next days, a gradual formation of the mechanical papillae has been observed. In the goose, four pairs of large conical papillae are formed in the shape of rounded primordia. In adult domestic geese, the first pair of the large conical papillae is composed of two parts differing in size: a larger lateral part and a smaller median part (Jackowiak et al. 2011). Our study showed that these papillae develop from one primordium which early undergoes division into lateral and medial parts. In domestic ducks at this stage, three pairs of rounded primordia of large conical papillae and three primordia of conical papillae of the lingual prominence are formed (Skieresz-Szewczyk et al. 2014a, b). Bryk et al. (1992) showed that in the chicken, on the border between the body and root of the tongue, the mucosa elevation in the form of a comb is created, on which at the end of the differentiation stage finger-like appendages develops.

The differentiation stage in birds corresponds to the embryonic period in mammals. Studies of the tongue development in mammals showed that during the embryonic period in the rat, mouse, and domestic cat, there are no primordia for mechanical papillae (Iwasaki et al. 1997a; 1999; Iwasaki and Asami, 2002). On the tongue of the rat, the primordia of the gustatory papillae, namely the fungiform papillae, appear (Iwasaki et al. 1997a).

The second stage of embryonic development, the growth stage in the goose, lasts from the 14th until the 25th day of incubation (39 HH stage to hatching). The existing four pairs of primordia of the large conical papillae, at the same time, change their shape and arrangement. Around the 16th day (40 HH stage), they are cone-shaped, and on 18th day (41/42 HH stage), all papillae are arranged at an angle of 20°–25° to the long axis of the tongue. During embryonic development, the height of the papillae increases about 15 times and the diameter increases about 4 times.

During the growth stage, 11 pairs of small conical papillae are formed, gradually in the direction towards the apex of the tongue. Changes in the arrangement of small conical papillae occur sequentially for each individual primordium. Just before hatching, only the first three pairs of small conical papillae have the shape of cones and are arranged at an angle of 30°. The measurements revealed that for the whole embryonic period, the height and diameter of the small conical papillae increase by only 60 μm. Around the 17/18th day (41/42 HH stage), the height of the papillae decreases, due to the fact that in these days, the embryonic epithelium is transformed into a multilayered flat epithelium built of 4–5 cell layers.

In that stage, on the medial part of all large conical papillae and the first pair of the small conical papillae of the body unique spherical structures were formed. These structures develop gradually on each successive pair of papillae from the 15th to 19th days of incubation (39/40 HH stage to 41/42 HH stage). Cross-sections through those structures show a mesenchymal core covered with epithelium. They are a part of the primordium of the papillae and not, as one might have expected, only epithelial thickenings. The time of appearance of these structures is followed by a change in the angle of inclination of the large conical papillae to the long axis of the tongue. It is difficult at the moment to explain that fact, but it may be assumed that these structures situated on the medial part of the papillae are involved in shifting the papillae nearer to the lingual body.

During the growth stage, development of ten conical papillae was observed on the caudal part of the lingual prominence. These papillae develop as separate mesenchymal elevations from the medial part of the lingual prominence symmetrically to its edges. The shape changing of these papillae, as in the case of the large conical papillae of the lingual body, also results from the process of papillae elongation, which starts around the 18th/19th day (41/42 HH stage). During the whole embryonic period, the height of the conical papillae of the lingual prominence increases four times and the diameter increases five times.

A study of Skieresz-Szewczyk et al. (2014a, b) in ducks revealed that during the growth stage, three more pairs of large conical papillae, 18–19 pairs of small conical papillae, and 13 more papillae of conical papillae of the lingual prominence in the first row and 12 papillae in the second row are formed. In the chicken, the finger-like papillae on the common crest are conical in shape (Bryk et al. 1992).

The comparative analyses of mechanical papillae development in birds revealed that conical papillae on the lateral edges of the tongue develop as separate elevations of the mesenchymal tissue successively in the direction towards the apex of the tongue, which results are connected with the elongation process of the lingual body (Skieresz-Szewczyk et al. 2014a, b). The pattern of development for conical papillae at the border between the body and the root of the tongue is different. In Anseriformes, conical papillae of the lingual prominence are formed as separate mesenchymal elevations in pairs, symmetrically on the right and left sides of the midline papillae (Skieresz-Szewczyk et al. 2014a, b). In Galliformes, a common mesenchymal primary crest develops from which conical papillae are gradually formed (Bryk et al. 1992).

The growth stage in birds corresponds to the fetal period in mammals. The mechanical papillae, referred to as filiform papillae, in the rat and mouse develop immediately before birth, and in the domestic cats, the hemispherical primordia of the filiform papillae are observed around the 30th day after conception (Tichy 1993; Iwasaki et al. 1997a, 1999; Iwasaki and Asami 2002).

In adult domestic geese, on the lateral sides of the lingual body, 11 pairs of small conical papillae and 4 pairs of large conical papillae are found (Jackowiak et al. 2011). Between the small and large conical papillae, filiform papillae are found, while on the caudo-lateral part of the body, there are hair-like papillae. On the caudal edge of the lingual prominence, ten conical papillae are present (Jackowiak et al. 2011).

This study revealed that at the end of the growth stage at the 25th day (45 HH stage) in goose embryos, all conical papillae develop but only the large conical papillae, the first 3 pairs of small conical papillae, and conical papillae of the lingual prominence have the same shape and arrangement as in adult birds. The remaining primordia of small conical papillae will probably change their shape and angle of inclination to the tongue after hatching. During the differentiation stage and the growth stage, the filiform papillae and hair-like papillae are not formed. The development of those papillae, which in fact are keratinized processes, begins at the moment of epithelium keratinization after hatching.

Based on microscopic observations of histogenesis of the epithelium covering mechanical papillae, we established three developmental stages such as the embryonic stage, transformation (growth) stage, and prehatching stage. Each developmental stage takes place on different days of incubation, depending on the area of the tongue and the type of the conical papillae.

The embryonic stage for the large conical papillae lasts from the 11th to 13th day, the small conical papillae from the 16th to 19th day, while for the conical papillae of the lingual prominence, it occurs at the 16th day of incubation. The epithelium on the conical papillae is composed of undifferentiated cells with microvilli on the surface.

The transformation (growth) stage for the large conical papillae lasts from the 14th until the 20th day, for the small conical papillae, from the 18th day until the end of the examined embryonic period, and for the conical papillae of the lingual prominence, it lasts from the 17th until the 22nd day. During this stage, epithelial cells change their shape and three layers: basal, intermediate, and superficial, may be distinguished. Additionally, the division of the intermediate layer into two parts, i.e., the upper and lower, is observed. The development of the epithelium layers is a sign of epithelial maturation towards the adult type epithelium.

Our observations also revealed the presence of characteristic granules in the cytoplasm of superficial cells in the epithelium covering conical papillae of the lingual prominence. In the available literature, similar granules, called periderm granules, have been reported only in the periderm, the protective layer of the epidermis, during the formation of skin and its keratinized appendages in reptiles and birds (Alibardi, 1998, 2002, 2009). Thus, we state that the superficial layer of the epithelium of the mechanical papillae in the domestic goose may be considered to be the periderm. The periderm during the embryonic development of the epidermis in mammals is the first protective barrier of the skin which, upon formation of a mature keratinized epithelium, undergoes desquamation and enters fetal slurry (Akiyama et al. 1999). It should be noted that in mammals during the formation of the keratinized layer of the mechanical papillae, in the cytoplasm of cells, granules appear, classified as keratohyalin granules, responsible for the development of the keratinized layer (Farbman 1970; Bragulla and Homberger 2009). Immunohistochemical studies in birds using antibodies—markers for filaggrin, a protein component of the keratohyalin granules in mammals, showed no presence of this protein in the granules observed in birds (Alibardi 2004).

The differentiating feature of epithelial cells at the embryonic and transformation stages in the domestic goose is the presence of microvilli on their surface. Bryk et al. (1992), and Skieresz-Szewczyk et al. (2014a, b) also observed the microvilli during tongue development. It seems, therefore, that microvilli are features of undifferentiated epithelial cells in the early stage of development.

The third stage, the prehatching stage, has been distinguished because of the formation of the keratinized layer of the epithelium on large conical papillae and on conical papillae of the lingual prominence. The keratinized layer is not formed on small conical papillae. On the basis of the microscopic observations, we may state that the keratinized layer on papillae develops in two different ways. The formation of the keratinized layer on large conical papillae occurs at the same time as the formation of the superficial layer. By contrast, on the conical papillae of the lingual prominence, the keratinized layer is formed after the disappearance of the embryonic superficial layer. Our observations of conical papillae of the lingual prominence also revealed that during the keratinization process of the epithelium, the initially visible condensed nuclei in the keratinized layer and specific granules in the superficial layer gradually disappear. A characteristic feature of superficial cells in the adult epithelium stage is the presence of number of microridges on their surface. Microridges, as membrane structures, are regarded as a characteristic describing differentiation in mature keratinocytes. Similar results were obtained during tongue development in the chicken and duck (Bryk et al. 1992; Skieresz-Szewczyk et al. 2014a, b).

Comparing the structural changes of conical papillae epithelium during embryogenesis in birds, we can state that the transformation begins approximately at two-thirds of the incubation period and the keratinization process begins immediately before hatching (Bryk et al. 1992; Skieresz-Szewczyk et al. 2014a, b). In mammals, the keratinization process also starts just before birth and continues in the postnatal period (Iwasaki et al. 1997a, b, 1999).

Referring to the obtained results concerning mechanical papillae development in the domestic goose to the research hypothesis, we can conclude that only large conical papillae of the lingual body and conical papillae of the lingual prominence are fully developed and have a similar structure to that in adult birds. Changes in the shape and arrangement of small conical papillae will probably continue after hatching, along with the formation of the keratinized layer. Similarly, filiform papillae and hair-like papillae develop after hatching.

Considering the function of the mechanical papillae in the goose, we state that the tongue at the time of hatching is well prepared for grazing using conical papillae of the lingual body, and it can gather food and prevent it from flowing back into the oral cavity using conical papillae of the lingual prominence. This finding explains the behavioral observation, which showed that birds aged several days actively cut the grass (Van der Leeuw et al. 2003). The absence of filiform papillae, which in adult birds form an important part of the filtration apparatus, correlates with previous findings of Van der Leeuw et al. (2003), who noticed that young birds can only peck food immersed in water. The fully mature mechanism of food filtration from water develops 2 weeks after hatching. It may be assumed that at that time filiform papillae develop and this process is initiated by the mechanical stimuli generated during food intake.

In order to accurately characterize the development of the mechanical papillae of the tongue in the domestic goose, in the future, it is planned to undertake studies after hatching. This research can determine the time and manner of development of filiform and hair-like papillae and describe changes of arrangement of small conical papillae and formation of their keratinized layer.
